# Inflammation: A Network in the Pathogenesis of Status Epilepticus

**DOI:** 10.3389/fnmol.2018.00341

**Published:** 2018-10-05

**Authors:** Ming Wang, Yinghui Chen

**Affiliations:** ^1^Department of Neurology, Jinshan Hospital, Fudan University, Shanghai, China; ^2^Department of Neurology, Huashan Hospital North, Fudan University, Shanghai, China

**Keywords:** status epilepticus, inflammation, cytokines, inflammatory signaling pathway, NF-κB

## Abstract

Status epilepticus (SE) is an abnormally prolonged or recurrent epileptic seizure that is a serious, life-threatening medical emergency. Notably, it requires prompt and aggressive treatment. SE is characterized by high mortality and morbidity. However, its pathogenesis remains unclear. Numerous studies of SE have reported widespread brain inflammation, suggesting that inflammation plays a vital role in the occurrence and development of SE. This mini review article reviews the current knowledge with regard to the role of inflammation in SE.

## Introduction

Status epilepticus (SE) is defined as an ongoing seizure or repetitive seizures within 5 min without a recovery to baseline clinical conditions between the seizures. Various neurological diseases, including brain trauma, brain infection, brain tumor and stroke may induce SE (Hocker, 2015; Trinka and Kälviäinen, 2017). Notably, SE is the second most common life-threatening neurological emergency, with a prevalence rate of 10–41 cases per 100,000 people (Betjemann and Lowenstein, 2015; Bragazzi et al., 2016). Despite the emergence of new antiepileptic drugs (AEDs) and surgery, the available therapies are frequently associated with severe side effects; furthermore, they are primarily symptom-driven and fail to address the underlying disease processes. Recent data show that seizures may be the result of an inflammatory reaction. Furthermore, evidence from clinical and experimental studies has demonstrated that pro-inflammatory pathways participate in the development of SE. Thus, it may be possible for experimenters to control seizures by modifying inflammatory signals in the brain (Vezzani and Friedman, 2011; Janigro et al., 2013).

## Inflammation and Status Epilepticus

Numerous studies have demonstrated that inflammation is not only a consequence of seizure activity but that it may also contribute to epileptogenesis. In this model, inflammation is induced by SE, which then exacerbates inflammation, and then further reduces the threshold of seizure onset, leading to a “vicious cycle” (Janigro et al., 2013). Although little is known in regard to the pathology of brain tissue in patients with SE, some clinical cases have been reported. For example, serum levels of interleukin-1β (IL-1β), IL-6, and high-mobility group box 1 (HMGB1) protein in children with febrile convulsions are elevated, compared with those in children with fever alone (Choi et al., 2011). Furthermore, higher levels of pro-inflammatory cytokines and chemokines have been found in the cerebrospinal fluid of refractory SE patients, compared with other patients with inflammatory neurological diseases (Sakuma et al., 2015). Finally, a clinical study found that approximately half of the patients with SE who were studied developed systemic inflammatory response syndrome (Szklener et al., 2017). Animal studies further support the following observations: induction of SE by chemical ignition or electrical stimulation can cause a rapid and intense inflammatory cascade in the brain, causing the activation of microglia and astrocytes. These activated microglia, reactive astrocytes and infiltrating immune cells release large amounts of pro-inflammatory mediators, including IL-1β, cyclooxygenase-2 (COX-2), HMGB1 and tumor necrosis factor α (TNF-α), which can induce neuroinflammation through a variety of signaling pathways. The inflammation can further increase excitability through neurogenesis, sprouting and neuronal damage, finally, leading to SE (Rojas et al., 2014; Dey et al., 2016; Srivastava et al., 2016). However, because of the complexity of the inflammatory cascade, the pathophysiology of various inflammatory molecules remains unclear. Here, we elaborate on the possible inflammatory response mechanisms in SE.

### NF-κB Signaling Pathway in SE

**Figure 1:**
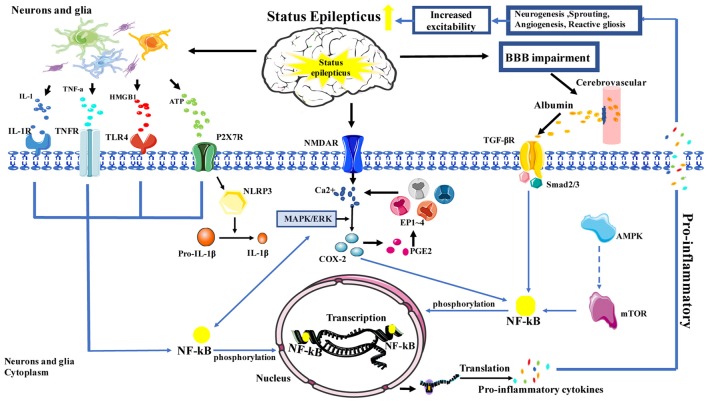
Schematic depicting the role of inflammation in status epilepticus (SE). Activation is represented by solid lines; inhibition is represented by dashed lines.

The NF-κB signaling pathway plays an important role in inflammation; notably, NF-κB is increasingly recognized as a key player in many steps of inflammation in SE. In the neuroinflammation process, NF-κB cooperates with a variety of other signaling molecules and pathways, such as COX-2, mammalian target of rapamycin (mTOR) and mitogen-activated protein kinase (MAPK). These signaling molecules and pathways can also interact with NF-κB and affect NF-κB target genes, which regulate NF-κB transcriptional activity, or affect upstream signaling pathways. In the classical NF-κB signaling pathway, HMGB1, TNF-α and IL-1 can activate toll-like receptor 4 (TLR-4), TNF receptor (TNFR) and IL-1R, respectively (Figure 1).

Previous studies have reported that NF-κB and SE are closely related. First, research showed that in animals without SE, NF-κB phosphorylation was rarely observed in the microglia; however, in rats with SE that was induced by LiCl-pilocarpine, NF-κB phosphorylation was apparently increased in the microglia (Shetty, 2011). Similarly, *in vitro* studies have shown low IL-1 and TLR expression in resting astrocytes; however, when seizures occur, astrocytes rapidly activate and release a large number of inflammatory factors (IL-1β, TNF-α), while simultaneously rapidly increasing the phosphorylation of NF-κB and the expression of IL-1R. Later, the IL-1β/IL-1R1 NF-κB signal induced inflammation leads to neurogenesis, sprouting, neuronal damage and death; these changes cause neuronal excitotoxicity that leads to SE (Vezzani and Friedman, 2011). Second, HMGB1, a protein that regulates gene transcription, is also related with SE. Moreover, studies have demonstrated that HMGB1 can activate the TLR-4/NF-κB signaling pathway. When seizures occur, HMGB1 is rapidly translocated from the cytoplasm and is released into the intercellular space through TLR-4. Subsequently, activated NF-κB signaling pathways promote the production of inflammatory mediators, in order to exert pro-inflammatory effects and to aggravate SE (Balosso et al., 2014). Besides, studies in pilocarpine-induced SE rat models showed that the expression of inflammation-related factors, such as MCP-1, TLR-4, and IL-6 in hippocampus and cerebral cortex, which were associated with the number of activated astrocytes and microglia cells can be downregulated by anti-HMGB1 mAb, which was associated with the number of activated astrocytes and microglial cells as well as the expression of IL-1β. The onset and latency of SE were significantly prolonged in the anti-HMGB1 mAb group (Fu et al., 2017). Furthermore, HMGB1 is a potential point of intersection between oxidative stress and inflammation, as HMGB1 promotes the production of reactive oxygen species (ROS), thereby, aggravating the inflammatory response (Pauletti et al., 2017). Lastly, TNF-α can activate both cell death and survival pathways; this balance ultimately determines whether TNF-α exerts neurodegenerative or neuroprotective effects (Tartaglia and Goeddel, 1992). A study in regard to SE reported that most of the activated microglia showed strong TNF-α immunoreactivity and TNF-α-associated signal transduction pathways, which involves the binding of NF-κB to TNFp75R, causing cell death; however, during the activation of p38 MAPK via downstream signaling, TNFp55R promotes neuronal survival time (Sriram and O’Callaghan, 2007). In addition to this, recent evidence suggests that TNF-α can be induced by purinergic ion channel receptor 7 (P2X7R) in SE; moreover, it can reduce neuronal damage via enhanced phosphorylation of NF-κB in the hippocampal CA3 region (Kim et al., 2011), suggesting that TNF-α may play a protective role in SE.

### mTOR Signaling Pathway in SE

mTOR is a serine-threonine kinase that senses the energy state of cells, and the mTOR signaling pathway is activated by a variety of stimulations. Several studies have shown that inhibiting the mTOR pathway can reduce seizures in SE models (Citraro et al., 2016), reduce the production of inflammatory factors, and restore the function of the blood-brain barrier (BBB; van Vliet et al., 2016). Furthermore, seizures can activate the mTOR pathway, increase the activity of NF-κB, and promote the expression and synthesis of inflammatory molecules, finally, leading to SE. Resveratrol or mTOR inhibitors can effectively inhibit NF-κB activation and reduce the production of inflammatory factors. Similarly, studies have shown that adenosine (ADO) can attenuate pentylenetetrazol-induced SE by inhibiting mTOR pathway via AMP-dependent protein kinase (AMPK; Wang et al., 2017). Therefore, the mTOR pathway plays an important role in the inflammatory pathway of SE.

### MAPK Signaling Pathway in SE

MAPKs comprise a group of enzymes with critical roles in the cellular response to various external stimuli; c-Jun-N-terminal kinase (JNK) is one of the members of this family. Recent studies have demonstrated that activation of the JNK pathway promotes the development of inflammation. Phospho-c-Jun can enter the nucleus and upregulate the expression of COX-2. In one study, it was reported that the absence of the *JNK1* gene had neuroprotective effects on the damage induced by SE (Busquets et al., 2018). In addition, leptomycin B can ameliorate SE-induced vasogenic edema via inhibition of the p38 MAPK pathway, indicating that the p38 MAPK signaling pathway may be involved in BBB disruption after SE (Kim et al., 2016), in a manner that is related to the inflammatory reaction.

### COX-2 and Prostaglandin E2 in SE

Recent studies have shown that the rapid and sustained expression of a large number of inflammation-related genes in SE is associated with elevated COX-2 protein, and COX-2 primarily plays an important role in the initiation or prolongation of the inflammatory reaction. Therefore, COX-2 is considered a driving force for seizure-induced inflammation (Jiang et al., 2015). In a mouse model of SE, COX-2 expression was significantly increased in hippocampal neurons within 1 h after seizure; the application of COX-2 inhibitors demonstrated an obvious therapeutic effect on SE. Prostaglandin E2 (PGE2) is a major product of COX-2 synthesis in the brain. PGE2 can bind to and activate four receptors, EP1, EP2, EP3 and EP4; increase Ca^2+^ release; and mediate various pathophysiological functions that lead to neuronal damage, which include plastic, neurologic deficit and hyperexcitability. Ultimately, reducing the seizure threshold to trigger SE, and SE through NMDA-induced neuronal damage increase Ca^2+^ release in cycle (Rojas et al., 2014). Recent evidence has shown that PGE2 mediates inflammation and brain damage mainly through EP2 receptors in SE (Du et al., 2016). Activation of EP2 receptors plays a dual role: early stimulation is associated with neuroprotection, whereas late stimulation increases neurotoxicity (Dey et al., 2016; Santos et al., 2017). In this context, pretreatment with EP2 receptor agonists can reduce SE. Notably, PE2 receptor antagonists accelerated body weight gain but reduced both cerebral inflammation and delayed-onset mortality in an SE mouse model, suggesting that PE2 receptor inhibition also inhibits inflammation and protects hippocampal neurons.

### P2X7R in SE

A member of the P2X family, P2X7R is a nonselective cation channel that is activated by extracellular ATP. During seizures, P2X7R can be activated by high concentrations of ATP, which consequently leads to the activation of microglia and triggers the release of IL-1β TNF-α, and PGE2 (Beamer et al., 2017). The P2X7R is also a potent stimulus for NF-κB pathways. But more importantly, P2X7R has a special role in the release of IL-1β that is involved in the activation of NLRP3 inflammasome (Di Virgilio et al., 2017). Increased P2X7R expression has been found in the cerebral cortex and the hippocampus of SE mice. After the administration of P2X7R inhibitor, reduced severity of epileptic seizures, microglial activation, IL-1β release and neuronal damage were observed. This suggests that P2X7R acts as a link between increased nerve excitability and neuroinflammation. Thus, it may become a new therapeutic target for the inhibition of inflammation and for seizure control (Henshall and Engel, 2015). In addition, a recent study with a rat model of SE found that blood-derived leukocytes were present in the brain parenchyma in an experimental group treated with ATP, suggesting that P2X7R regulates the infiltration of leukocytes in SE through neurotransmission and glial transfusion. Notably, this provides a new treatment target for SE (Kim et al., 2010), while further uncovering the role of P2X7R-related inflammatory signaling pathways in SE.

### TGF-β Signaling Pathway in SE

Abnormal BBB function is the main cause of irregular neuronal discharge. Recent studies have shown that SE can cause BBB damage and increase its permeability (van Vliet et al., 2016); this permits immune cells, inflammatory mediators and albumin to infiltrate the brain parenchyma, causing inflammatory reactions. Similarly, BBB breakdown has been confirmed as the most common pathological change in SE patients, occurring in up to 55% of the cases (Malter et al., 2018). Exogenous inflammatory mediators that enter the brain parenchyma change the sensitivity of the ion channels, reducing the seizure threshold, affecting the absorption and release of neurotransmitters, and increasing the excitability of neurons (Srivastava et al., 2016). Recent studies have shown that albumin exuded by BBB dysfunction activates the TGF-β signal (Cacheaux et al., 2009), which then activates NF-κB, resulting in increased neuronal excitability and neuronal damage. Transforming growth factor β is a pleiotropic cytokine that plays a key role in cell communication and participates in growth, differentiation and immune response. Studies have shown that TGF-β is upregulated in the hippocampus of those with SE and is translocated to the nucleus through the phosphorylation of the Smad2/3 protein complex. It then regulates transcriptional activation, participates in BBB destruction and microglial activation (induced by peripheral cells), causes inflammation, and ultimately leads to nerve injury, thus, aggravating SE (Vezzani et al., 2013).

## Conclusion

SE can cause damage to the BBB and activate a variety of cells, including microglia, astrocytes, monocyte-macrophages and neutrophils. Subsequently, activated microglia, reactive astrocytes, endothelial cells of the BBB and the infiltrating immune cells release large amounts of pro-inflammatory mediators, including IL-1β, IL-6, COX-2, HMGB1, TNF-α and chemokines, which can induce neuroinflammation through a variety of signaling pathways. Furthermore, numerous studies have shown that signaling pathways downstream of NF-κB and their targets modulate the production of inflammatory cytokines, ultimately, modulating neuroinflammation. This neuroinflammation can further aggravate the severity, duration, or frequency of SE, leading to a “vicious cycle.” Therefore, NF-κB plays an important role in this complex network of inflammatory responses (Figure 1) and may provide a novel endogenous therapeutic target for neuroinflammation in SE. However, an important limitation that needs to be considered is that inflammation may be a minor factor or simply a consequence of SE. Other factors that were found to influence SE have been explored in several studies, which include infection, autoimmune diseases and other brain disease (Sakuma et al., 2015; Caputo et al., 2018); further studies are required to clarify this.

## Author Contributions

MW and YC conceived the article and wrote the manuscript. YC reviewed and edited the manuscript. All authors read and approved the manuscript.

## Conflict of Interest Statement

The authors declare that the research was conducted in the absence of any commercial or financial relationships that could be construed as a potential conflict of interest.
